# Hospitals’ Age-Friendly Health Systems Recognition and 30-day Mortality Following Hip Fracture Surgery

**DOI:** 10.1093/geroni/igaf122.3602

**Published:** 2025-12-31

**Authors:** Hiroshi Gotanda, Teryl Nuckols, Brandon Truong, Zhaohong Feng, Carol Lin, Kathleen Breda, Mark Vrahas

**Affiliations:** Cedars-Sinai Medical Center, Los Angeles, California, United States; Cedars-Sinai Medical Center, Los Angeles, California, United States; Cedars-Sinai Medical Center, Los Angeles, California, United States; Cedars-Sinai Medical Center, Los Angeles, California, United States; Cedars-Sinai Medical Center, Los Angeles, California, United States; Cedars-Sinai Medical Center, Los Angeles, California, United States; Cedars-Sinai Medical Center, Los Angeles, California, United States

## Abstract

Hip fractures among older adults present complex clinical challenges that extend far beyond surgical repair. Age-Friendly Health Systems (AFHS) recognition—a geriatric care initiative created to advance evidence-based, interdisciplinary geriatric care, with formal recognitions beginning in 2019—offers a promising pathway to better hip-fracture outcomes. However, the national impact of this initiative on outcomes among older adults with hip fracture remains largely unknown. In this context, we sought to compare 30-day postoperative mortality rates between older adults with hip fracture treated at acute care hospitals with AFHS recognition vs. those treated at hospitals without such recognition. We identified Medicare fee-for-service beneficiaries 65 years and older who underwent hip fracture surgeries based on relevant Current Procedural Terminology codes using 2021 Medicare data. We determined AFHS recognition status using the AFHS database. We fitted a multivariable linear regression model adjusted for patient characteristics. Our study included 27,201 observations (across 2,764 hospitals), 1,964 (7.2%) of which were treated at 141 hospitals with AFHS recognition. We found no evidence that 30-day postoperative mortality rates differ between hospitals with vs. without AFHS recognition (adjusted mortality rate, 6.7% vs. 7.5%; adjusted difference, -0.7 percentage points; 95% confidence interval, -1.7 to + 0.3; P-value = 0.17). While the difference was not statistically significant, the magnitude may present a clinically meaningful difference. Further multi-year, methodologically robust studies are needed to assess the national impact of the AFHS recognition initiative on outcomes among older adults with hip fracture and guide health policy, including CMS’s Age-Friendly Hospital Measure beginning in 2025.

